# Specificity of Methylation Assays in Cancer Research: A Guideline for Designing Primers and Probes

**DOI:** 10.1155/2010/870865

**Published:** 2010-07-27

**Authors:** Zeinab Barekati, Ramin Radpour, Corina Kohler, Xiao Yan Zhong

**Affiliations:** Laboratory for Gynecological Oncology, Women's Hospital and Department of Biomedicine, University of Basel, Basel 4031, Switzerland

## Abstract

DNA methylation is an epigenetic regulation mechanism of genomic function, and aberrant methylation pattern has been found to be a common event in many diseases and human cancers. A large number of cancer studies have been focused on identification of methylation changes as biomarkers (i.e., breast cancer). However, still clinical use of them is very limited because of lack of specificity and sensitivity for diagnostic test. This highlights the critical need for specific primer and probe design to avoid false-positive detection of methylation profiling. The guideline and online web tools that are introduced in this paper might help to perform a successful experiment and to develop specific diagnosis biomarkers by designing right primer pair and probe prior to experimental step.

## 1. Introduction

DNA methylation of cytosine located 5′ to a guanosine is one of the most important modifications of genomic DNA in eukaryotic cells. Methylation of cytosine at CpG dinucleotides is described as an epigenetic regulation mechanism of genomic function that plays an important role in different biological processes including embryogenesis [1], genomic imprinting [2], X-chromosome inactivation, and cancer [3, 4].

Aberrant methylation pattern has been found to be a common event in many cancers [5–7]. Global hypomethylation is considered to play a role in carcinogenesis; however, local hypermethylation changes gene expression [8]. This hypermethylation alteration resulted in transcriptional inactivation followed by silencing of promoter at nearby tumor suppressor genes, contributing to development of cancer. The hypermethylation was thought to be an early event in carcinogenesis [9–12]. A large number of studies in cancers including breast cancer have focused on the use of CpG island hypermethylation profiling as cancer biomarkers in tissue and circulating cell-free DNA of patients, with the aim of improving cancer treatment via accurate early diagnosis, noninvasive diagnosis, prognosis, and prognosis therapy selection [7, 13–18].

Recent technology development has provided the analysis of DNA methylation in a genome-wide scale [19, 20] which may not be easily accessible for many institutions. Thereby, in most of the research centers methylation assays can be only determined on gene-by-gene-based methods that use bisulfite conversion. The bisulfite reaction was first described in early 1970s [21, 22]. Since the first description of bisulfite reaction in the application of studying CpG sites, many methods based on the same principle have been developed and categorized according to primer designing strategies. Based on primer designing strategies two different DNA methylation assays are described, methylation-independent-specific PCR (MIP) primers and methylation-specific PCR (MSP) primers [23].

Primer and probe design for methylation assays based on bisulfite conversion is challenging because of the DNA composition after bisulfite modification. One of the most critical steps for methylation study is designing primers and probes for the modified DNA and it needs special constrains on primers or probe and their location on the DNA. A large number of studies have been focused on identification of biomarkers; however, the clinical use of these biomarkers is still very limited because of lack of specificity and sensitivity for diagnostic test. This highlights the critical need for specific primer and probe design to avoid false-positive detection of methylation.

We review a brief guideline of CpG island prediction, designing primers and probes for MIP and MSP assays that are used for methylation studies based on bisulfite conversion. Some important web-tools for methylation studies are introduced as well.

## 2. CpG Island Prediction

Methylation at the cytosine bases of CpG dinucleotide-rich region mostly within 0.5–4 kb are known as CpG islands [24, 25]. Although analysis of the methylation status of some critical CpG sites as biomarkers are better than others, it is essential to find CpG islands at the promoter region of candidate genes which are in close proximity to the transcription start site.

In order to predict CpG islands as target region, the following rules should be applied.

- If CpG island prediction is used for primer design and more than one island is found, any of the predicted islands can be a target region for primer selection.
- If a CpG island size is smaller than the minimum product size, the primer pair should span the whole island.
- If a CpG island size is greater than the maximum product size, the primer pair should be within the island.
- If a CpG island size is between the minimum and maximum product size, at least two thirds of the island region should be amplified.

## 3. Methylation-Independent-Specific PCR (MIP) Primers

MIP primers are used in different PCR-based methylation analysis methods including bisulfite-sequencing PCR (BSP) (in 1992, [26]), pyrosequencing [27, 28], combined bisulfite restriction analysis (COBRA) [29], methylation-sensitive single-nucleotide primer extension (MS-SnuPE) [30–32], methylation-sensitive melting curve analysis (MS-MCA) [33], methylation-sensitive high-resolution melting (MS-HRM) [34], matrix-assisted laser desorption/ionization time-of-flight (MALDI-TOF) mass spectrometry with base-specific cleavage and primer extension [35, 36], heavy methyl [37], and microarray DNA methylation profiling technique based on bisulfite conversion, that is, methylation-specific oligonucleotide microarray (MSO) [38].

Incomplete bisulfite modification of DNA is sometimes a concern [39] and results in high representation of methylation levels in studied samples. Successful application of MIP methods depends on whether PCR primer could be designed to amplify the complete modified fragment of interest. To reduce bias of bisulfite-modified DNA against unmodified or incompletely modified DNA or even unsuccessful experimental PCR optimization, primer pair should be picked from a region that have adequate number of cytosines “C”s (no-CpG) in the original sequence [40]. Primer pairs with more “C”s will be preferred by receiving higher weighing scores and increasing the annealing temperature (Table 1). Besides general consideration for designing primer pair, the following constraints are enforced for MIP primer design.

**Figure 1:**
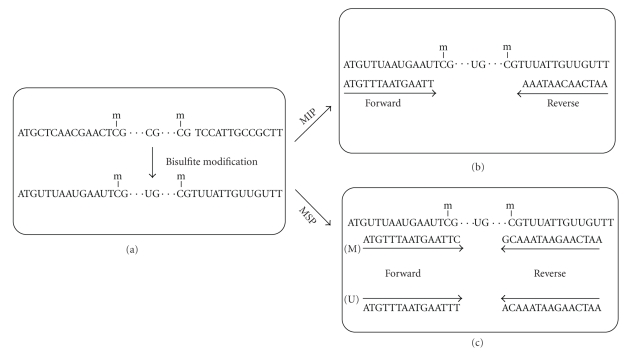
Primer design for DNA methylation profiling techniques based on bisulfite conversion. (a) First DNA is treated with sodium bisulfite to convert all unmethylated cytosines to uracil. To analyze DNA methylation status of the interest genes, converted DNA is amplified based on two different primer designing strategies: methylation-independent specific PCR (MIP) and methylation-specific PCR (MSP). (b) In MIP, DNA molecules are amplified using primer pairs containing cytosines (no-CpG) in their sequence. (c) In MSP, primer pairs are designed to specifically amplify either methylated (M) or unmethylated (U) DNA by containing CpG site in their sequence that makes possible to distinguish the methylated sequence from the unmethylated sequence.

- Primers should not contain any CpG sites within their sequence to avoid discrimination against methylated or unmethylated DNA (Figure 1).
- Primers should have an adequate number of “C”s (no-CpG) in their sequence to amplify only bisulfite modified DNA. Primers with more “C”s will be preferred (at least 30%) [40] (Figure 1).
- A good primer pair should span a maximal number of CpG sites in the selected amplicon to map as many CpG sites as possible.
- If CpG island prediction is not used for primer selection, selected amplicons must span at least 5 CpG sites as a default.
- Long length primer (25–30 mer) is preferred to ensure uniqueness of the primer [39].
- Primer sets should not amplify more than 500 bp because DNA degradation occurs by bisulfite modification.

## 4. Methylation-Specific PCR (MSP) Primers

Methods based on MSP primers are considered to have the highest analytical sensitivity and are designed to specifically amplify either methylated or unmethylated DNA by using primers that distinguish the methylated sequence from the unmethylated sequence [23, 40]. The precision and sensitivity of MSP depends on appropriate primer or probe design not prone to false-positive results [23]. MSP primers-based methods include methylation-specific PCR (MSP) [40], methylight [41, 42], SYBER green-based quantitative MSP [43, 44], sensitive melting analysis after real-time MSP (SMART-MSP) [45], and methylation-specific fluorescent amplicon generation (MS-FLAG) [46]. The specificity of methylation-based PCR methods is achieved by appropriate primer pair or probes design (Table 1). The following constraints are recommended to reduce false-priming events for amplification of methylated DNA.

- To discriminate between a methylated and unmethylated DNA fragment, primers have to contain as much CpG sites as possible (at least one CpG) preferably at the very 3′-end. At least one of the last three bases at 3′-end of the primer has to be a CpG “C” (Figure 1).
- A part from CpG site(s) at the very 3′-end, additional CpG sites in a primer sequence is preferred (Figure 1).
- Primers for methylated DNA and unmethylated DNA should contain the same CpG sites in their sequence. For example, a forward primer for methylated pair has this sequence: ATAAGTATTCGTTAATGGTTCGA, the forward primer in the unmethylated pair must also contain the two CpG sites, for example, ATAAGTATTTGTTAATGGTTTGA. But they may differ in length and start position [3].
- The two sets of primers for methylated and unmethylated DNA should have similar *T*_*m*_ values (max *T*_*m*_ difference 5°C).
- Elimination of secondary structure formation and primer-dimer pairs by increasing primer length.
- Primer sets should not amplify more than 500 bp because DNA degradation occurs by bisulfite modification.

False-priming event can be prevented by designing appropriate primers and increasing annealing temperature. Having an appropriate negative control in the experiment might help to find out false-priming events.

## 5. Guidelines for Probe Designing

In methylation studies, the discrimination between methylated and unmethylated DNA is achieved by three ways: design of primers that contain or does not contain CpG sites, design of fluorescent labeled probe (for instance MSO and bead array), and design of the both primer and probe, that is, methylight technology [41]. MIP and MSP methods are associated with false positive results. By using fluorescent probes, for instance methylight methodology or applying heavy methyl probe-based methodology, the false positives can be limited. Using probe as a detection method increases the specificity to discriminate between methylated and unmethylated DNA by designing probes that contain additional CpG sites [40]. The selection of new primer pairs for methylation-specific PCR and suitable hybridization probes for real-time PCR-based assays require the identification of the CpG sites that are methylated (Table 1). Moreover, using probe provides possibility to detect more than one target with multiplex reaction by different reporter dyes [38, 47].

In addition probe-based assays can provide quantitative information; further advantages are the speed and high throughput of the 96-well-based, real-time PCR system and the omission of all postamplification steps, which has less labour and the risk of contamination. Also, the efficiency of individual reactions is accessible from the slope of the amplification plot in the logarithmic phase. This allows for the direct quality control of every amplification reaction and the identification of samples containing impurities or poor template that interfered with optimal amplification and thereby with the quantification [48].

A general guideline for probe designing is described as follows:

- The probe sequences should include 3 to 5 potential methylation sites to maximize specificity and reduce false-priming event.
- The probe binding sites should include several cytosines in the original sequence to ensure specificity for converted DNA and overcome false positives due to incomplete bisulfite conversion.
- Long repetitive stretches should be avoided.
- Probe Tm value should be 10°C higher than primers.
- G + C content should be 30%–80%.
- No G should be at the 5′ end.
- Probes should have 15–30 mer in length.
- No more than two G + C should be at the 3′ end.
- Amplicon size should be 50–150 bp (max 300 bp).The PCR products should be as short as possible, to maximize efficiency (especially important for the analysis of fragmented DNA isolated from formalin-fixed, paraffin-embedded biopsies, and circulating cell-free DNA).

## 6. Online Web Tools for Methylation Study

### 6.1. DNA Methylation Analysis Databases

- Entrez Gene: (http://www.ncbi.nih.gov/entrez).
- GDB: Human Genome Database (http://www.gdb.org/).
- DNA methylation database: public resource to store and standardise DNA methylation data (http://www.methdb.de/).
- methBLAST: similarity search program designed to explore in silico bisulfite modified DNA, either or not methylated at its CpG dinucleotides (http://medgen.ugent.be/methBLAST/).
- DNA Methylation Society: an international scientific society open to all those interested in any aspects of biological methylation (http://www.dnamethylation.net/).

### 6.2. Promoter Prediction Tools

- FirstEF: first-exon and promoter prediction program for human DNA (http://rulai.cshl.org/tools/FirstEF/).
- Promoter 2.0 Prediction Server: Promoter 2.0 predicts transcription start sites of vertebrate PolII promoters in DNA sequences (http://www.cbs.dtu.dk/services/Promoter/).
- WWW Promoter Scan: predicts Promoter regions based on scoring homologies with putative eukaryotic Pol II promoter sequences (http://thr.cit.nih.gov/molbio/proscan/).
- McPromoter MM: The Markov Chain Promoter Prediction Server. McPromoter is a program aiming at the exact localization of eukaryotic RNA polymerase II transcription start sites (http://genes.mit.edu/McPromoter.html).

### 6.3. CpG Island Prediction Tools

- CpG Island Searcher (http://cpgislands.usc.edu/).
- CpG Plot (http://www.ebi.ac.uk/emboss/cpgplot/).
- MethPrimer (http://www.urogene.org/methprimer/).
- CpGProD (CpG Island Promoter Detection): CpGProD is a mammalian-specific software which proposes to identify the promoter regions associated with CpG islands (CGIs). CpGProD uses the structural characteristics of the CGIs associated with promoters (start CGIs). In the first step, CpGProD searches for all the CGIs located over the sequences and, in the second step, CpGProD identifies start CGIs and orientation of the potential promoters (http://pbil.univ-lyon1.fr/software/).
- CpG island Explorer for local installation (http://www.hku.hk/).

### 6.4. Methylation PCR Primer Design Tools

- MethPrimer: CpG island prediction, MSP, MSI primer design. By using this software 5′ and 3′ ends of primer pair should have sites where conversion has occurred (C to T). This is to avoid amplification bias towards the unconverted sequence (http://www.urogene.org/methprimer/).
- BiSearch: BSP and MSP primer design (http://bisearch.enzim.hu/).
- PerlPrimer: PerlPrimer is a free, open-source application written in Perl that designs primers for standard PCR, bisulfite PCR, real-time PCR (QPCR) and sequencing. It aims to automate and simplify the process of primer designing (http://perlprimer.sourceforge.net/).
- BiQ Analyzer: software tool for easy visualization and quality control of DNA methylation data from bisulfite sequencing (http://biq-analyzer.bioinf.mpi-inf.mpg.de/).

### 6.5. Methylation BLAST (metthBLAST)

methBLAST (http://medgen.ugent.be/methBLAST/) is a sequence similarity search program designed to explore *in silico* bisulfite modified DNA (either or not methylated at its CpG dinucleotides) to provide a search portal for validated methylation assays. The tool is mainly developed to find primer binding sites and hence addresses specificity for PCR-based assays that use bisulfite converted DNA as input material, including bisulfite sequencing, methylation-specific PCR, COBRA, bisulfite-PCR-SCCP (BiPS), Ms-SNuPE, and PCR melting curve analysis.

## 7. Discussion

The large number of investigations such as human epigenome project (HEP) and cancer studies focused on DNA methylation analysis based on bisulfite modification provided valuable information about methylation variable positions that might influence genes activity (http://www.epigenome.org) [7, 16, 49, 50]. Increasing knowledge about methylation status of genes involved in carcinogenesis can lead to discovering new biomarkers that could be used for early detection, management, diagnosis or therapeutic approaches in cancer patients. Developing biomarkers by methylation analyzing methods requires accuracy, sensitivity, low-false-positive and false-negative rates and high-throughput evaluation of single CpG sites. Although different useful technologies exist for methylation assessment, no method is universal. While besides choosing a method according to type of samples and possessed laboratory special equipment, right choice of CpG island and primer or probe will minimize the risk of failed experiment.

Right primer and probe design is crucial for successful PCR amplification of bisulfite-modified DNA. Bisulfite reaction not only causes the expected conversion of cytosines to uracils, but also causes undesired DNA strand breakage. Loss of DNA during the subsequent purification step is another concern especially when studying microdissected DNA samples. All these factors pose challenges to downstream PCR applications and primacy of designing primers and probe for such PCR-based assays. Mostly, amplification of a product size greater than 500 bp is difficult after bisulfite-modified DNA template; hence, it might be better to set the default product size range as 100–500 bp for primer design. Another option that differs from standard PCR is primer length. Bisulfite conversion-based PCRs generally require longer primers. Primers with a length of approximately 30 mer usually yield successful results [39]. The reason is that bisulfite modification decreases considerably GC content of DNA templates and produces long stretches of “T”s in the sequence that makes it difficult to pick primers with acceptable Tm values or stability. In other words, in order to discriminate modified DNA and unmodified or incompletely modified DNA, enough number of “C”s is required in primers and probes, which makes picking stable primers more demanding. Thus, to achieve better duplex stability, choosing longer primer is necessary as *T*_*m*_ of DNA. In practice, size of primers for such PCR-based assays usually ranges from 20 to 30 mer [3, 40, 51].

Much more effort is needed to validate an experiment for clinical use of biomarkers such as easy to use method, sensitivity and specificity, appropriate primers and probes, easily interpretable results, and cost-effectiveness. The guidelines and the online web tools that are introduced in this review might help to have a successful experiment and to develop specific diagnosis biomarkers by designing right primer pair and probe prior to experimental step.

## Figures and Tables

**Table 1:** The main characteristics for primer/probe designing in DNA methylation profiling techniques based on bisulfite conversion.

Primer/Probe	Main characteristics
MIP primer	(i) No CpG sites within the sequence.
	(ii) Including an adequate number of “C”s (no-CpG) in the sequence.
	(iii) Spanning a maximal number of CpG sites in the amplicon.
	(iv) Long length primer (25–30 mer).
	(v) Amplicon size maximum 500 bp.

MSP primer	(i) Containing as much CpG sites as possible especially at 3′-end of the primer.
	(ii) Considering the same CpG sites in the primer sequence for methylated DNA and unmethylated DAN primers.
	(iii) Similar Tm values for both the methylated DNA and unmethylated DAN primers.
	(iv) Amplicon size maximum 500 bp.

Probe	(i) Including CpG sites to maximize specificity.
	(ii) Including several “C”s (no-CpG) in the sequence.
	(iii) Probes length 15–30 mer.
	(iv) Amplicon size 50–150 bp (max 300 bp).
